# Linking the Phytochemicals and the α-Glucosidase and α-Amylase Enzyme Inhibitory Effects of *Nigella sativa* Seed Extracts

**DOI:** 10.3390/foods10081818

**Published:** 2021-08-06

**Authors:** Salima Tiji, Mohamed Bouhrim, Mohamed Addi, Samantha Drouet, Jose Manuel Lorenzo, Christophe Hano, Mohamed Bnouham, Mostafa Mimouni

**Affiliations:** 1 Laboratory of Applied Chemistry and Environment (LCAE), Faculty of Sciences Oujda (FSO), University Mohammed First (UMP), Oujda 60000, Morocco; mimouniosrn@gmail.com; 2 Laboratory of Bioresources, Biotechnology, Ethnopharmacology and Health, Faculty of Sciences Oujda (FSO), University Mohammed First (UMP), Oujda 60000, Morocco; mohamed.bouhrim@gmail.com (M.B.); mbnouham@yahoo.fr (M.B.); 3 Laboratoire d’Amélioration des Productions Agricoles, Biotechnologie et Environnement (LAPABE), Faculté des Sciences, Université Mohammed Premier, Oujda 60000, Morocco; m.addi@ump.ac.ma; 4 Laboratoire de Biologie des Ligneux et des Grandes Cultures, INRA USC1328, Orleans University, CEDEX 2, 45067 Orléans, France; samantha.drouet@univ-orleans.fr; 5 Centro Tecnológico de la Carne de Galicia, Rúa Galicia Nº 4, Parque Tecnológico de Galicia, San Cibrao das Viñas, 32900 Ourense, Spain; jmlorenzo@ceteca.net; 6 Área de Tecnología de los Alimentos, Facultad de Ciencias de Ourense, Universidad de Vigo, 32004 Ourense, Spain

**Keywords:** *Nigella sativa* L., seeds, phytochemical, acute toxicity, antidiabetic activity, intestinal α-glucosidase, pancreatic α-amylase

## Abstract

*Nigella sativa* L. (*Ranunculaceae*), commonly referred to as black seeds or black cumin, is used in popular medicine (herbal) all over the world for the treatment and prevention of several diseases, including diabetes. This study aims to investigate the inhibitory effect of *N. sativa* extracts and fractions against the activities of intestinal α-glucosidase and pancreatic α-amylase in vitro, and to explain the inhibitory effect of these fractions against these enzymes by identifying their active compounds responsible for this effect and determine their modes of inhibition. To do so, *N. sativa* hexane and acetone extracts were prepared and analyzed by GC–MS and HPLC–DAD, respectively. The hexane extract was further fractioned into eight different fractions, while the acetone extract generated eleven fractions. The extracts as well as the resulting fractions were characterized and evaluated for their potential in vitro antidiabetic activity using intestinal α-glucosidase and pancreatic α-amylase inhibitory assays in vitro. Hexane extract and fractions were less active than acetone extract and fractions. In the case of intestinal α-glucosidase activity, the acetone fraction SA3 had a high inhibitory effect on intestinal α-glucosidase activity with 72.26 ± 1.42%, comparable to the effect of acarbose (70.90 ± 1.12%). For the pancreatic α-amylase enzymatic inhibitory assay, the acetone fractions showed an inhibitory capacity close to that for acarbose. In particular, the SA2 fraction had an inhibitory effect of 67.70 ± 0.58% and was rich in apigenin and gallic acid. From these fractions, apigenin, (−)-catechin, and gallic acid were further characterized for their inhibitory actions. IC_50_ and inhibition mode were determined by analyzing enzyme kinetic parameters and by molecular modeling. Interestingly, (−)-catechin showed a possible synergistic effect with acarbose toward α-glucosidase enzyme inhibition, whereas apigenin showed an additive effect with acarbose toward α-amylase enzymatic inhibition. Furthermore, we studied the toxicity of *N. sativa* hexane and acetone extracts as well as that of acetone fractions. The result of acute toxicity evaluation demonstrated that *N. sativa* extracts were nontoxic up to a concentration of 10 g/kg, except for fraction SA3. Taken together, these results indicate that *N. sativa* extracts and/or derived compounds could constitute promising nutraceuticals for the prevention and treatment of type 2 diabetes mellitus.

## 1. Introduction

Diabetes mellitus (DM) has been rising at an unprecedented rate around the world. DM is a severe, chronic, and multifaceted metabolic disease with serious repercussions, including long-term disruption, failure, and dysfunction of various vital organs [1,2]. The number of people living with DM is steadily rising, and by 2035, it is expected to exceed nearly 600 million (World Health Organization, 2021). Type 2 DM (T2DM) is the most common type of diabetes, accounting for about 90% of all diabetes cases (World Health Organization, 2021). T2DM is characterized by hyperglycemia, decreased glucose tolerance, and insulin resistance and hyperlipidemia, and is caused by the inefficient use of insulin [2]. T2DM is primarily exacerbated by excess body weight and inactivity, and it is linked to a lower quality of life as well as an elevated risk of death and morbidity (World Health Organization, 2021). It has a significant economic impact on individuals, families, health systems, and countries (World Health Organization, 2021).

In diabetic patients, postprandial hyperglycemia is involved in the glycation of plasma and cellular proteins, which contributes to the development of diabetes complications. In this regard, the management of diabetes mellitus requires accurate postprandial glycemic control by decreasing glucose absorption. This is possible via the inhibition of carbohydrate enzymes [3].

Intestinal α-glucosidase (EC3.2.1.20) and pancreatic α-amylase (EC3.2.1.1) are essential enzymes for carbohydrate digestion and absorption and have been identified as effective therapeutic targets for modulating the pathologic postprandial hyperglycemia detected in T2DM patients [1,2]. Pancreatic α-amylase is responsible for important steps in starch digestion, resulting in linear maltose and branched isomaltose oligosaccharides. These oligosaccharides are then digested further by intestinal α-glucosidase resulting in the release of absorbable monosaccharides. In this sense, the use of inhibitors of intestinal α-glucosidase and/or pancreatic α-amylase can effectively slow the digestion and assimilation of starch at the early stages of digestion, resulting in a substantial delay in postprandial hyperglycemia and a favorable impact on insulin resistance and glycemic index regulation [2].

Clinically, drugs such as acarbose, voglibose, or miglitol are used for this purpose. However, these drugs often induce serious gastrointestinal side effects including stomach pain, flatulence, and diarrhea [4]. As a result, natural α-glucosidase and α-amylase inhibitors, mostly from food products, have emerged as promising therapeutic options to supplement or even substitute existing drugs [2,5,6,7]. Several types of natural plant products have emerged as promising α-glucosidase and α-amylase inhibitors in recent decades [8,9,10,11,12,13,14,15,16].

Black cumin (*Nigella sativa* L.) is an annual herb from the *Ranunculaceae* family. Its black seeds have been used in traditional medicine for anticancer, analgesic, antidiabetic, anti-inflammatory, antihypertensive, antimicrobial, antioxidant, and immunomodulatory purposes [17,18,19,20,21]. Oil from black cumin seed, obtained after cold pressing, rich in terpenes, sterols, and tocopherols, has substantial applications in cosmetics, cooking, and pharmacy [21,22]. The resulting seed cake is also rich in phenolic compounds, which can be valued in a variety of nutraceutical or cosmetic applications [23].

Several studies have shown that *N. sativa* may have antidiabetic properties [18,19]. *N. sativa* seed extracts were shown to regulate hyperglycemia and enhance diabetes management in a variety of animal models, with a substantial decrease in fasting and 2 h postprandial blood glucose levels, a decrease in glycated hemoglobin, improved insulin tolerance, and an increase in serum insulin [18,19]. In particular, in rats, it demonstrated a rise in insulin and C-peptide levels as well as a normalized glycemia [19]. Furthermore, *N. sativa* seed lipids administered at 4% in streptozotocin (STZ)-induced diabetes in rats resulted in a reduction in toxicology [24]. In addition, in humans, administering *N. sativa* extract increased ACC phosphorylation, resulting in insulin sensitization [25]. The majority of these studies were used on *N. sativa* extracts, but isolating active fractions or identifying active compounds from these extracts would allow for the attribution and rationalization of the antidiabetic behavior of *N. sativa*.

That is why this study aims to investigate the inhibitory effect of *N. sativa* extracts and fractions against the activities of intestinal α-glucosidase and pancreatic α-amylase in vitro, and to explain the inhibitory effect of these fractions against these enzymes by identifying their active compounds responsible for this effect, determining their modes of inhibition, and evaluating their toxicity. The identification and characterization of the compounds were carried out by GC–MS and HPLC–DAD in comparison with local database abs standards. Inhibitory actions of potential antidiabetic compounds were further characterized by the determination of their IC_50_ values and inhibition modes both by analyzing enzyme kinetic parameters and by molecular modeling.

## 2. Materials and Methods

### 2.1. Chemicals

All solvents and products of analytical grade (99.8%), silica gel, acarbose, apigenin, (−)-catechin, gallic acid, intestinal α-glucosidase, and pancreatic α-amylase were purchased from Sigma Aldrich (Saint-Quentin Fallavier, France).

### 2.2. Extracts and Fractions Preparation

**Figure 1:**
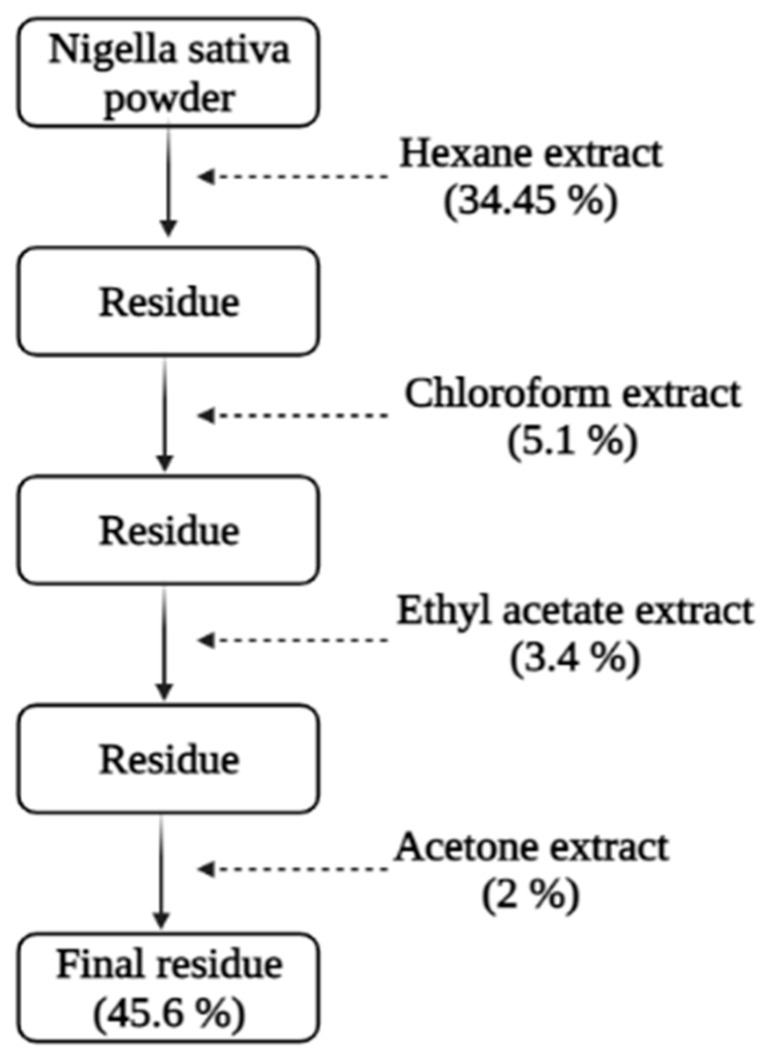
Successive extractions of *N. sativa* L. seeds.

*N. sativa* seeds were purchased from a local organic market (produced locally Oujda, Morocco, 34°41′21″ N 1°54′41″ W, July 2020). The seeds were washed before being ground into a fine, homogeneous powder. This powder was then placed in the Soxhlet apparatus and extracted at 50 °C in general, simultaneously, using hexane and acetone (Figure 1). The solvents from the extracts were removed using a vacuum rotatory at 40 °C, and the dried extracts were stored at −4 °C until use.

The hexane and acetone extracts were fractioned by column chromatography (6.5 cm × 47 cm) using high-purity grade silica gel column (pore size: 60 Å 63–200 µm) at ambient temperature and standard pressure. The solvent eluents of hexane and acetone extract separation were dichloromethane and cyclohexane with (2/8) and (5/5) *v*/*v* ratios, respectively.

### 2.3. Chemical Characterization

#### 2.3.1. GC–MS Analysis

Hexane and acetone extracts were analyzed by gas chromatography coupled to mass spectroscopy using Shimadzu (Kyoto, Japan) GCMS-QP2010. The column dimensions were (30 m × 0.25 mm, 0.25 µm) and the mobile phase used was helium gas. Ionization temperature was maintained at 200 °C during an analysis time of 28 min an electron ionization source with a single-quadrupole mass analyzer (Shimadzu, Kyoto, Japan). Identification was possible by comparing fragments mass and retention time to standards through the computer library NIST147.LIB [26].

#### 2.3.2. HPLC–DAD Analysis

Acetone extract and fractions were analyzed by HPLC using a Waters (Milford, MA, USA) e2695 HPLC system analysis with a C18 column that has (5 µm, 250 mm × 4.6 mm) dimensions. Eluents were water/acetic acid (2% *v*/*v*) (A) and acetonitrile, pH = 2.6 (B). The injection volume was 30 μL. The separation was done on gradient mode and the flow was maintained to 0.9 mL/min as previously described [27]. The detection was done using a diode array detector set during analysis on a 280–360 nm interval. Compounds were identified based on a comparison of chromatogram retention time and λmax with authentic standards.

### 2.4. The Effect of N. sativa Seed Extracts and Fractions on the Activity of Digestive Enzymes

It should be noted that the optimal dose (i.e., that resulting in at least 30% inhibition and applied at a concentration of no more than 1 mg/mL) was first determined using serial dilution (data not shown), and then both this concentration and half of this concentration were used to evaluate for each in a vitro inhibition assay.

#### 2.4.1. In Vitro Intestinal α-Glucosidase Inhibitory Assay

The effect of the *N. sativa* seed extracts and fractions against intestinal α-glucosidase activity was quantified colorimetrically by monitoring the glucose release from sucrose degradation, according to the protocol described by Ouassou et al. [28]. The assay mixtures contained 100 µL of sucrose (50 Mm), 1000 μL of phosphate- buffer (50 mM; pH = 7.5), and 100 μL of intestinal α-glucosidase enzyme solution (10 IU). Then, 10 μL of distilled water (control), acarbose (positive standard drug control), or *N. sativa* seed extracts or fractions solutions at two different concentrations (166 and 328 µg/mL) were added to the mixture. Then, tubes were incubated at 37 °C in a water bath for 25 min. The mixture was heated at 100 °C for 5 min to stop the enzymatic reaction, and the release of glucose was estimated by the glucose oxidase method using a commercially available auto-kit. The absorbance was measured at 500 nm, and the inhibition percentage was calculated using the below formula:

Inhibitory activity percentage = ((A_control 500 nm_ − A_Test 500 nm_)/A_control 500 nm_) × 100 (1)

A_Control 500 nm_: Absorbance of enzymatic activity without inhibitor.

A_Test 500 nm_: Absorbance of enzymatic activity in the presence of *N. sativa* extracts or fractions, or acarbose.

#### 2.4.2. In Vitro Pancreatic α-Amylase Inhibitory Assay

The inhibition of pancreatic α-amylase activity by *N. sativa* seed extracts and fractions was studied according to the procedure described by Daoudi et al. [29]. The assay mixtures contained 200 μL of pancreatic α-amylase enzyme solution (13 IU), 200 μL of phosphate-buffer (0.02 M, pH = 6.9) and 200 μL of *N. sativa* extracts and fractions at two different concentrations (0.5 and 1 mg/mL) or acarbose (positive standard drug control; 0.5 and 1 mg/mL). The mixtures were pre-incubated at 37 °C for 10 min. Then, 200 μL of starch (1% (*w*/*v*)) dissolved in phosphate buffer was added to each tube and were incubated for 20 min at 37 °C. To stop the enzymatic reaction 600 μL of DNSA color reagent was added. Hereafter, the tubes were incubated for 8 min at 100 °C, before being put in an ice-cold-water bath for a few minutes. The mixture was diluted by adding 1 mL of distilled water and the absorbance was measured at 540 nm. Inhibition percentage was calculated using the formula bellows:

Inhibitory activity percentage= ((A_Control 540 nm_ − A_Test 540 nm_)/A_Control 540 nm_) × 100 (2)

A_Control 540 nm_: Absorption of enzymatic activity without inhibitor.

A_Test 540 nm_: Absorption of enzymatic activity in the presence of *N. sativa* extracts or fractions, or acarbose.

### 2.5. Inhibition Mechanism Analysis

#### 2.5.1. IC_50_ Determination

To calculate the IC_50_ value, various concentrations (from 10 to 100 µM for apigenin, (−)-catechin and gallic acid, and from 1 to 10 µM for acarbose) were used. The assays were conducted as described above. The concentration of each inhibitor causing the 50% activity inhibition was calculated from % activity vs inhibitor concentration curves using software ED50 plus v1.0 (Mexico, Mexico). The experiments were repeated three times. Fractional inhibitory concentrations (FICs) at the IC_50_ level were calculated for both potential inhibitors, as follows: FIC = IC_50_ drugs in combination with acarbose/IC_50_ potential inhibitor alone.

#### 2.5.2. Enzyme Kinetic Parameters Determination

The enzyme assay was conducted as described above using increasing concentrations of substrate (15–50 mM for intestinal α-glucosidase; 10–100 mM for pancreatic α-amylase) in presence of inhibitors (apigenin and gallic acid for intestinal α-glucosidase; (−)-catechin and gallic acid for pancreatic α-amylase) used at 50 µM final concentration. Acarbose was used as a commercial drug at 5 µM. Lineweaver–Burk double reciprocal representation (plotting 1/velocity vs 1/substrate concentration) was used to assess inhibition mode, and kinetic parameters Km and Vmax were determined using the Michaelis-Menten equation.

Secondary plots were used to establish the inhibition constants Ki (slope of the Lineweaver–Burk double reciprocal representation vs inhibitor concentration and intercept of the Lineweaver–Burk double reciprocal representation vs inhibitor concentration).

#### 2.5.3. Molecular Docking Analysis

Docking data for the binding of apigenin and gallic acid with pancreatic α-amylase, (−)-catechin, and gallic acid with α-glucosidase were performed with PyRx virtual screening tool software, which includes Autodock 4 and Autodock Vina (The Scripps Research Institute, La Jolla, CA, USA) and Pymol v2.1.1 (Schrodinger, New York, NY, USA) to predict the conformation of these molecule ligands within the appropriate target binding site of pancreatic α-amylase (PDB: 2QMK) and α-glucosidase (PDB: 5NN5). The software Discovery Studio 2020 (Dassault Systemes, Vélizy-Villacoublay, France) was used to determine the type of interaction and visualization in 2D, and UCSF Chimera 1.14 (San Francisco, CA, USA) was used for the 3D representation of molecules and interaction residues. The docking protocol employed was described by Proença et al. [10] for α-glucosidase and Proença et al. [11] for α-amylase, but using human pancreatic α-amylase (PDB: 2QMK) and α-glucosidase (PDB: 5NN5). The 3D structure of each ligand was retrieved from PubChem (available online: https://pubchem.ncbi.nlm.nih.gov/ (accessed on 25 March 2021)). Initial virtual screen with the whole enzyme was conducted with the following sizes of the grid box: 81 Å × 82 Å × 85 Å (for α-glucosidase) and 60 Å × 78 Å × 63 Å (for α-amylase) in the *x*, *y,* and *z* dimensions, respectively, to identify the most favorable binding site predicted by the program based on the lowest docking energy and maximum docking number. The docking was then refined using this site with a grid box of 25 Å (square).

### 2.6. Acute Toxicity

#### 2.6.1. Experimental Animals

The present study was conducted by using Swiss *albinos* mice from the local animal husbandry department of the Faculty of Science, Mohammed First University (Oujda, Morocco). The animals were grouped in polycarbonate cages with soft bedding and *ad libitum* water and food access in an environmentally controlled room (22–26 °C, with a 12/12 h photoperiod). All mice were cared for in compliance with the internationally accepted guide for the care and use of laboratory animals published by the U.S. National Institutes of Health [30].

#### 2.6.2. Oral Acute Toxicity in Mice

Acute plant extracts’ toxicity was evaluated orally using albino mice (22–32 g). Thirty mice, after fasting for 16 h, were sorted into five groups (*n* = 6; ♂/♀ = 1 each) for each plant extract and fraction. *N. sativa* extracts were administered orally at single doses of 1, 3, 5, 7, and 10 g/kg body weight, respectively, while the control group received 10 mL/kg of distilled water. *N. sativa* fractions were administered orally, at single doses of 0.1, 0.3, 0.5, and 0.7 g/kg of body weight. The signs of toxic effects and/or mortality were observed continuously after 2 h and every 24 h for 14 days after administration.

### 2.7. Statistical Analysis

Data are presented as the mean ± standard errors and were subjected to statistical analysis using Graph Pad Prism 5.04 software (San Diego, CA, USA). Multiple-group comparisons were analyzed by one-way analysis of variance (ANOVA). Statistical significance was accepted as *p* ≤ 0.05.

## 3. Results and Discussion

### 3.1. Extracts and Fractions Characterization

#### 3.1.1. Extraction Yields

Hexane and acetone are polar and lipophilic solvents, respectively, with major logP differences. Hexane is a nonpolar organic solvent with a high lipophilia (logP = +3.9), allowing it to extract only hydrophobic compounds. Acetone is a polar solvent with a logP = −0.16 that is closer to the lipophilic–hydrophilic boundary line than that of hydrophobic compounds, meaning that it can solubilize hydrophilic compounds much better than hydrophobic compounds. The aim of using these two solvents with such a large lipophilia difference is to isolate and separate compounds with very different physicochemical properties.

*N. sativa* hexane extract (EH) was a brown liquid, whereas acetone extract (EA) had a thick caramel color and consistency. Yields calculated referring to *N. sativa* seeds weights. The highest yield was obtained with hexane (34.2% (*w*:*w*)), while a lower yield was obtained with acetone (2.0% (*w*/*w*)). Eight fractions (SH1–SH8) were obtained from EH, and eleven fractions (SA1–SA11) were obtained from EA. Except for fractions SA3, SA5, and SA11, the majority of fractions were yellow (SA11). The fraction SA3 was orange, the fraction SA5 was transparent, and the fraction SA11 was brownish.

#### 3.1.2. GC–MS Characterization

**Figure 2:**
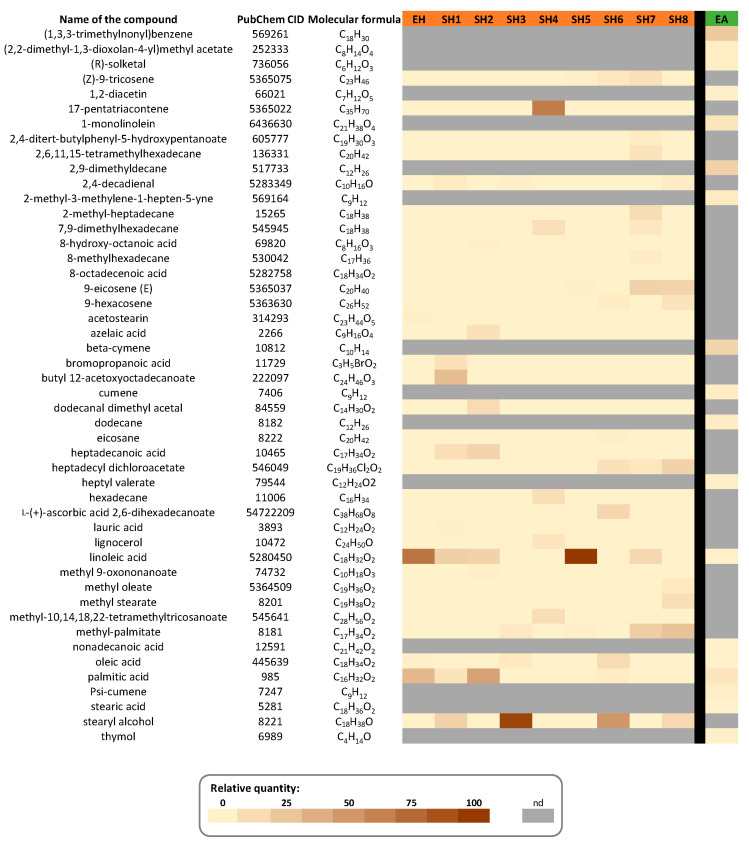
Relative abundance (in % of TIC (total ion chromatogram)) of the main components of *N. sativa* seeds hexane (EH) and acetone (EA) extracts and resulting hexane fractions (SH1-SH8) determined by GC–MS analysis. GC–MS chromatograms are shown in Figure S1. Values are given in Table S1. nd: not detected.

The presence of four major components from EH, predominantly fatty acids, was established using gas chromatography coupled to mass spectrometry (GC–MS) analysis: linoleic acid (64.52%), palmitic acid (30.33%), oleic acid (2.74%), and acetostearin (2.40%) (Figure 2; Figure S1a; Table S1). The occurrence of both unsaturated (linoleic (18:2 *cis*-9,12) and oleic (18:1 *cis*-9)), in higher amounts, and saturated (palmitic (16:0)) acids is consistent with the composition of *N. sativa* [31,32,33]. Acetostearin is a well-known functional ingredient derived from *N. sativa* oil that is used in the food industry or as an emollient in cosmetics [34].

#### 3.1.3. HPLC–DAD Characterization

**Figure 3:**
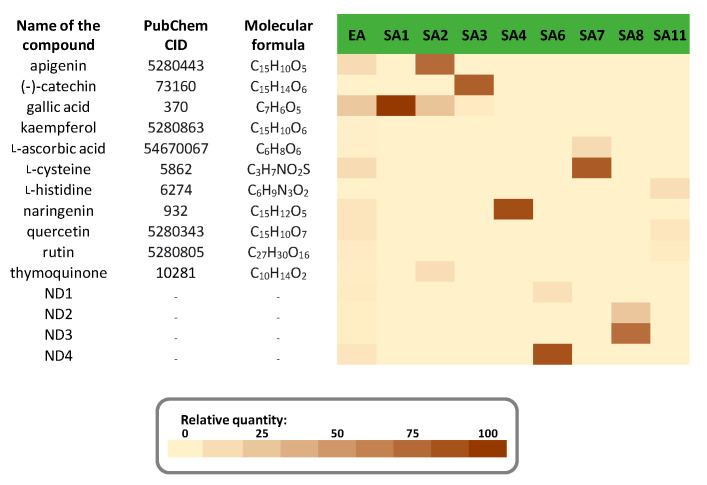
Relative abundance (in % of total peak area) of the main components of the *N. sativa* seeds acetone extract (EA) and its resulting acetone fractions (SA1–SA11) determined by HPLC–DAD analysis. HPLC–DAD chromatograms are shown in Figure S2. UV spectra are shown in Figure S3. Values are given in Table S2.

Considering their more hydrophilic nature, acetone extract (FA) and fractions (SA1-SA11) were characterized using high-performance liquid chromatography coupled to diode array detection (HPLC–DAD) (Figure 3; Figures S2–S4; Table S2).

This analysis revealed the presence of thymoquinone, phenolic compounds (including flavonoids (apigenin, kaempferol, quercetin, rutin, naringenin, and (−)-catechin) and gallic acid), amino acids (l-cysteine and l-histidine), and l-ascorbic acid. These compounds were partitioned into different fractions. Gallic acid was the most abundant component in fraction SA1, but it was also found in fractions SA2 and SA3. Apigenin was the main component of fraction SA2, which also included thymoquinone. Fraction SA3 was primarily composed of (−)-catechin, fraction SA4 of naringenin, and fraction SA7 of l-cysteine, with a trace of l-ascorbic acid. The last fraction with identified compounds, SA11, reassembled rutin, quercetin, and l-histidine. Fractions SA6 and SA8 were examples of fractions composed of four unidentified compounds, as were fractions SA9 and SA10 (data not shown). The phenolic content of *N. sativa* seeds was reported to be about 3% (*w*/*w*) of *N. sativa* seeds, which was in strong accordance with our acetone extraction yield [35]. Here, the acetone extract characterization results were quite similar to those reported by Mechraoui et al. [27]. Thymoquinone is one of the most prominent constituents of *N. sativa* seeds [36]. The presence of flavonoids [37,38], phenolic acids [39], and amino acids [40], in particular, have previously been found in *N. sativa* seeds.

### 3.2. The Effect of N. sativa Seed Extracts and Fractions on the Activity of Digestive Enzymes

#### 3.2.1. In Vitro Intestinal α-Glucosidase Inhibition Activity

**Figure 4:**
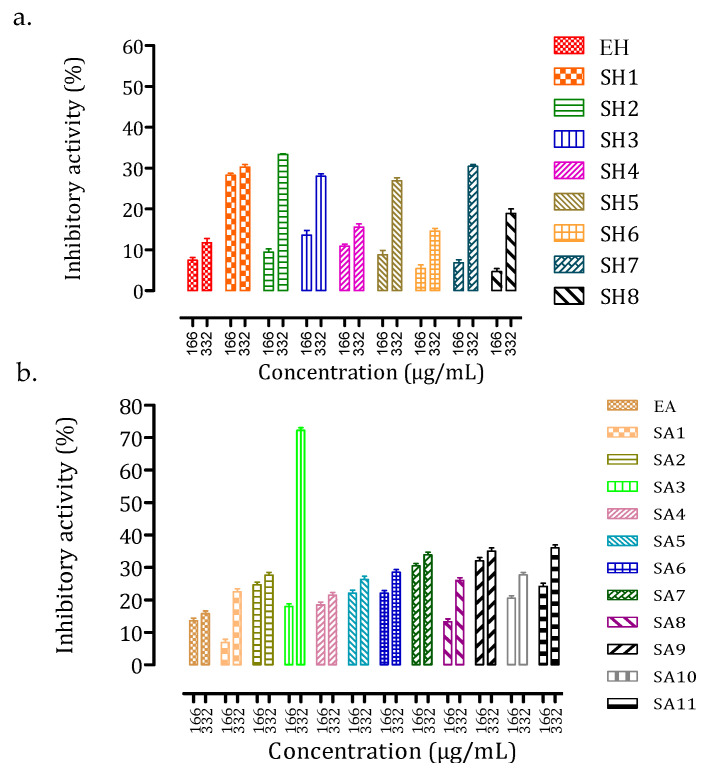
Inhibition of intestinal α-glucosidase by *N. sativa* seed (**a**) hexane extract (EH) and resulting fractions (SH1–SH8) and (**b**) acetone extract (AE) and resulting fractions (SA1–SA11) at two doses (166 and 332 µg/mL). The drug acarbose was used as a positive control at 50 µM (3.22 µg/mL) and resulted in 89.2 ± 0.5% inhibition. Extraction solvent was used as negative control (blank) and was subtracted to each corresponding extract or fraction inhibition value. Data are mean values ± standard deviation of 3 independent experiments.

The inhibitory capacity of each extract and fraction, at two doses (166 and 332 µg/mL), against intestinal α-glucosidase activity was determined (Figure 4).

For mother extracts EH and EA, only a minor inhibition of intestinal α-glucosidase activity was observed (11.76 ± 1.72% and 15.84 ± 1.42%, respectively). With the concentrations of extract or fraction added, the inhibition values generally rose, indicating the presence of at least one inhibitor of this enzyme in the considered extract/fraction. Acetone fractions showed a slightly higher inhibition compared to hexane fractions, with inhibition percentages ranging from 14.60 ± 1.12% (SH6) to 33.37 ± 0.31% (SH2) for hexane fractions and from 21.44 ± 1.54% (SA1) to 72.26 ± 1.42% (SA3) for acetone fractions. Polyphenolic compounds were more abundant in acetone fractions than in hexane fractions. The most active acetone fraction, SA3, was particularly rich in (−)-catechin and gallic acid. Several phenolics, including gallic acid and (−)-catechin, have been found to inhibit intestinal α-glucosidase, which supports this observation [8,9,10,41,42,43,44].

#### 3.2.2. In Vitro Pancreatic α-Amylase Inhibition Activity

**Figure 5:**
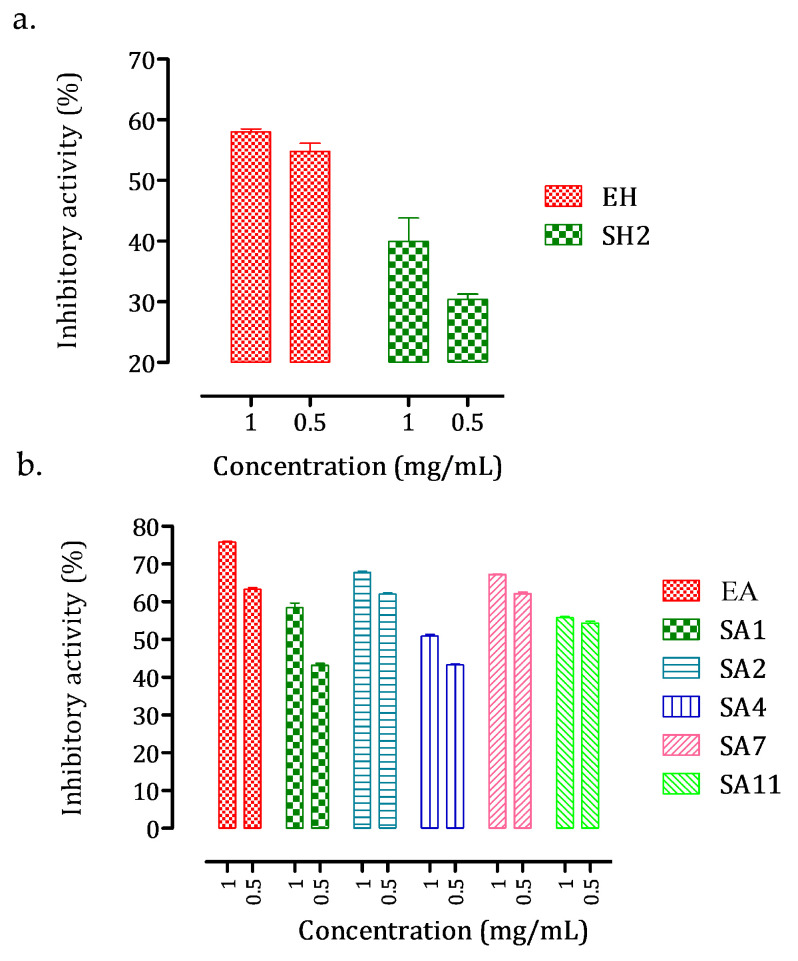
Inhibition of pancreatic α-amylase by *N. sativa* seed (**a**) hexane extract (EH) and resulting fractions (SH2) and (**b**) acetone extract (AE) and resulting fractions (SA1–SA11) at two doses (0.5 and 1.0 mg/mL). The drug acarbose was used as a positive control at 5 µM (3.22 µg/mL) and resulted in 65.6 ± 4.1% inhibition. Extraction solvent was used as negative control (blank) and was subtracted to each corresponding extract or fraction inhibition value. Data are mean values ± standard deviation of 3 independent experiments.

Inhibitory capacity against pancreatic α-amylase was also determined for each extract and fraction at two doses (0.5 and 1.0 mg/mL) (Figure 5).

The inhibition of acetone extract (EA, 75.8 ± 0.36%) was greater than that of hexane extract (EH, 58.0 ± 10.86%) and very similar to that of the standard drug acarbose (at 5 µM). Only the fraction SH2 showed substantial inhibition (39.9 ± 6.7%) among the various hexane fractions examined, albeit at a lower level than the mother hexane extract (EH). This suggests that the inhibitory activity is certainly the result of synergy and that separating the hexane extract is not beneficial for pancreatic α-amylase inhibition. Acetone fractions, on the other hand, were significantly more active than hexane extracts, except for fractions SA3, SA6, and SA8, which were inactive against pancreatic α-amylase activity. The highest inhibition was obtained for fractions SA2 (67.70 ± 0.58%) and SA8 (67.22 ± 0.24%). According to HPLC–DAD analysis, fraction SA2 was rich in apigenin and gallic acid, while fraction SA8 had two unidentified compounds. The identification and characterization of these two compounds will be the focus of future studies. As with intestinal α-glucosidase, some experiments suggest that apigenin and gallic acid could be involved in this inhibition of pancreatic α-amylase [8,9,11,12,13,14,15,16,45], most likely in combination with at least one of the two unidentified fraction SA8 compounds.

#### 3.2.3. Mechanism and IC_50_ Determinations

**Figure 6:**
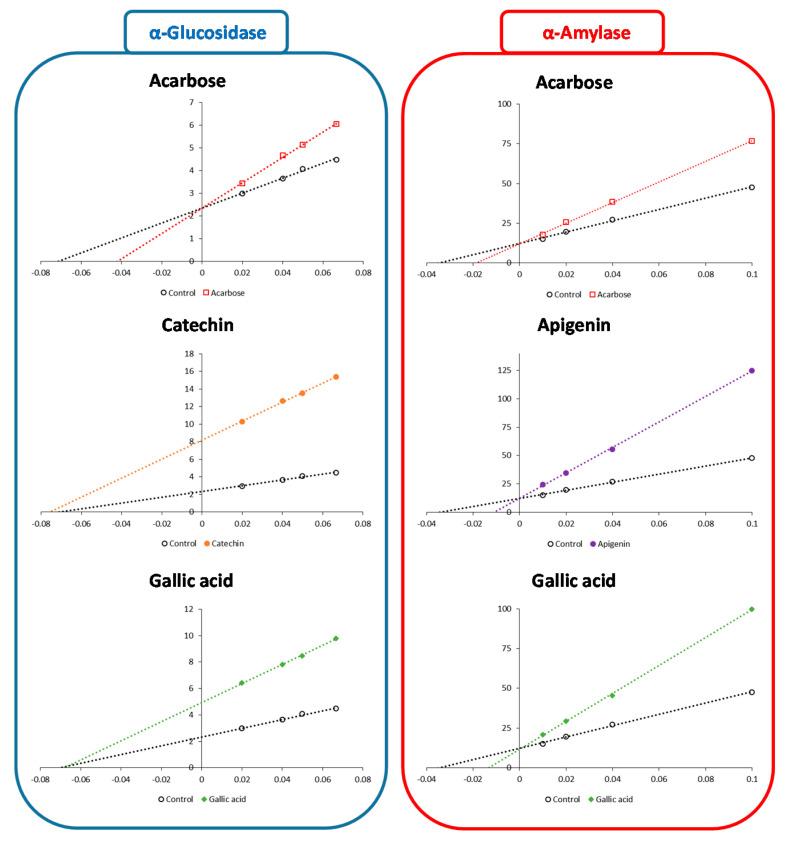
Lineweaver-Burk plots (with *x* = 1/v (v: velocity, in µmol/L); *y* = 1/[S] ([S]: substrate concentration in µM)) for inhibition of intestinal α-glucosidase by (−)-catechin (50 µM), gallic acid (50 µM), and acarbose (5 µM) and of pancreatic α-amylase by apigenin (50 µM), gallic acid (50 µM), and acarbose (5 µM).

The evolution of the kinetic parameters of intestinal α-glucosidase in the presence of (−)-catechin, gallic acid, and thymoquinone (50 µM), and of pancreatic α-amylase in the presence of apigenin and gallic acid (50 µM), was investigated and compared to the action of acarbose (5 µM). These kinetic parameters were determined from Lineweaver–Burk plots (Figure 6).

The results showed effective inhibition of intestinal α-glucosidase by both (−)-catechin and gallic acid, whereas thymoquinone was not active under the evaluated concentration range (data not shown). Similarly, both apigenin and gallic acid were effective inhibitors of pancreatic α-amylase. Contrary to acarbose, which acts as a competitive inhibitor (an increase of Km), Lineweaver–Burk plots indicated that (−)-catechin and gallic acid inhibit intestinal α-glucosidase in a noncompetitive manner by lowering its Vmax (Table 1). The Lineweaver–Burk plots for pancreatic α-amylase, like that for acarbose, indicated a competitive inhibition (increases of Km) by apigenin and gallic acid.

Given that both enzymes are hydrolases with very similar catalytic mechanisms and substrate binding cavities, this result may appear surprising. It is worth noting that acarbose inhibits both enzymes using the same mechanism, which is understandable given that its structure resembles that of an oligosaccharide [10,11]. However, despite their structural similarity, quercetin and taxifolin (dihydroquercetin) have been shown to inhibit α-glucosidase in different ways, with competitive inhibition for quercetin and noncompetitive inhibition for taxifolin [10]. The only difference between the two compounds is that taxifolin lacks the C2 = C3 double bond that quercetin has. However, the absence of C2 = C3 double bonding causes the B-ring to migrate out of the plane of the A- and C-rings, destroying its overall planarity, which thus may negatively impacting its binding capabilities [10]. Similarly, it has been found that quercetin and its glycoside form rutin and inhibit α-amylase with distinct inhibition modes: noncompetitive inhibition for quercetin vs competitive inhibition for rutin [46]. Both compounds can bind the active site (i.e., potential competitive inhibition), although with differing affinities, according to a molecular docking study (with higher affinity for rutin). The existence of the glycoside moiety in rutin, according to the authors of this study, is not the only factor responsible for this particular behavior; the compound size is also responsible. Because the cavity of the active site is large enough to accommodate polysaccharides, the authors supposed that smaller molecules cannot bind as well in the active site as larger molecules, as rutin, being larger than quercetin, could demonstrate [46]. These two cases also revealed that quercetin has different inhibitory modes with a noncompetitive inhibition of α-amylase vs a competitive inhibition of α-glucosidase [10,46].

**Figure 7:**
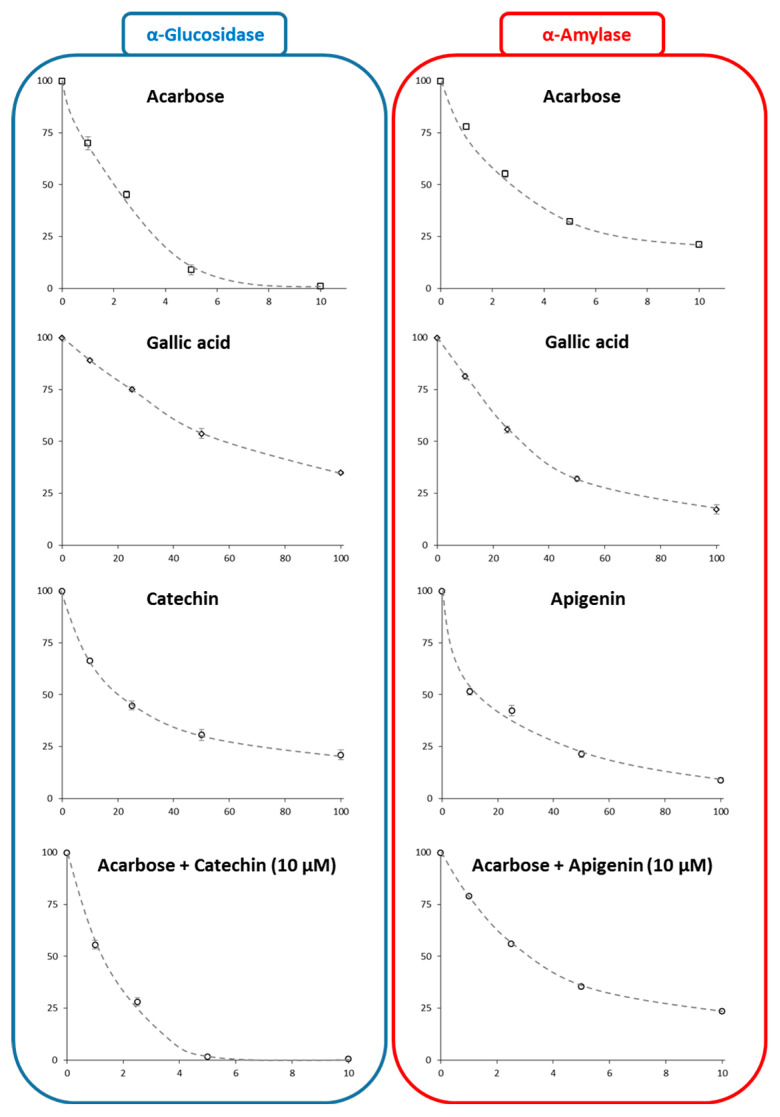
IC_50_ curves (with *x* = inhibitor concentration in µM; *y* = inhibition percentage) for inhibition of intestinal α-glucosidase by (−)-catechin, gallic acid, and acarbose alone or in the presence of (−)-catechin (10 µM), and pancreatic α-amylase by apigenin, gallic acid, and acarbose alone or in the presence of apigenin (10 µM).

The IC_50_ values were calculated from IC_50_ plots (Figure 7, Table 2). (−)-catechin was found to be more effective than gallic acid at inhibiting intestinal α-glucosidase activity, and when mixed with acarbose, it presented a synergistic effect (fractional inhibition concentration (FIC) = 0.16). Similarly, apigenin was found to be more effective than gallic acid at inhibiting pancreatic α-amylase activity and showed a possible additive effect when combined with acarbose (FIC = 0.74). These results are in good agreement with literature data [9,10,11,12,13,47], but more importantly support the traditional use of *N. sativa* seed to manage DM [17,18,19]. Interestingly, these results also indicated that *N. sativa* seed extracts, or purified (−)-catechin or apigenin, could be used in combination with acarbose to treat T2DM without reducing the effectiveness of this drug.

#### 3.2.4. Molecular Docking

**Figure 8:**
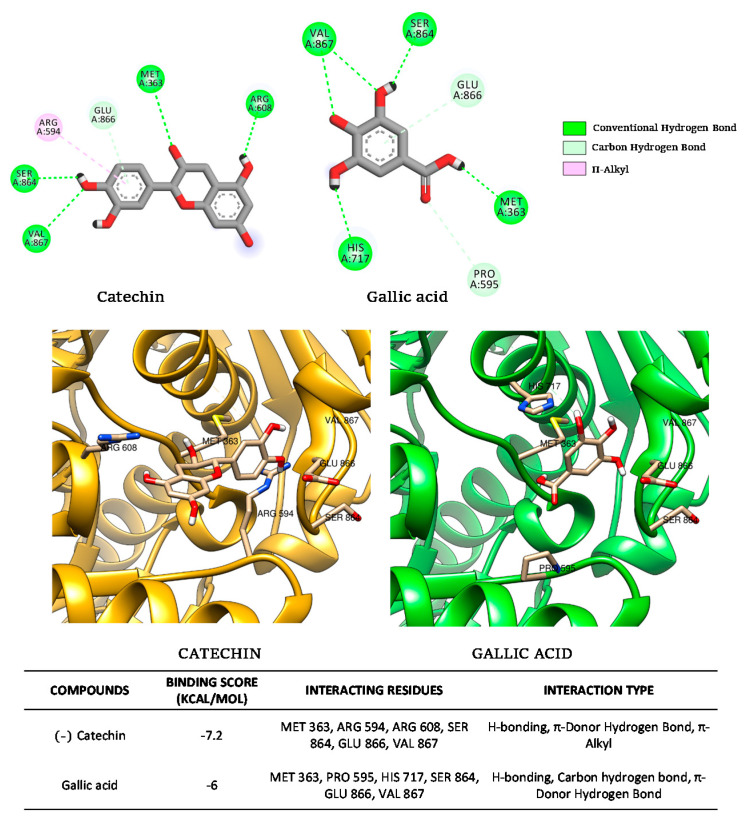
Molecular docking simulations of binding of (−)-catechin and gallic acid to human intestinal α-glucosidase.

Figure 8 presents the docking results made for (−)-catechin and gallic acid for their possible binding to intestinal α-glucosidase.

These results confirmed the binding of both compounds to intestinal α-glucosidase outside the active site, thus confirming their noncompetitive action. The calculated affinities were −7.2 kcal/mol and −6.0 kcal/mol for (−)-catechin and gallic acid, respectively, thus confirming our experimental enzymatic data. This difference in favor of (−)-catechin can be the consequence of more stable interactions as observed with an additional π-alkyl interaction (with R594 of the enzyme). Other conventional hydrogen bonds (for example, with S864 and M363) and carbon–hydrogen bonds with E866 were observed for both (−)-catechin and gallic acid. Similarly, Zhang et al. [9] also reported the noncompetitive binding of flavonoids such as bacalein to α-glucosidase with similar affinities.

**Figure 9:**
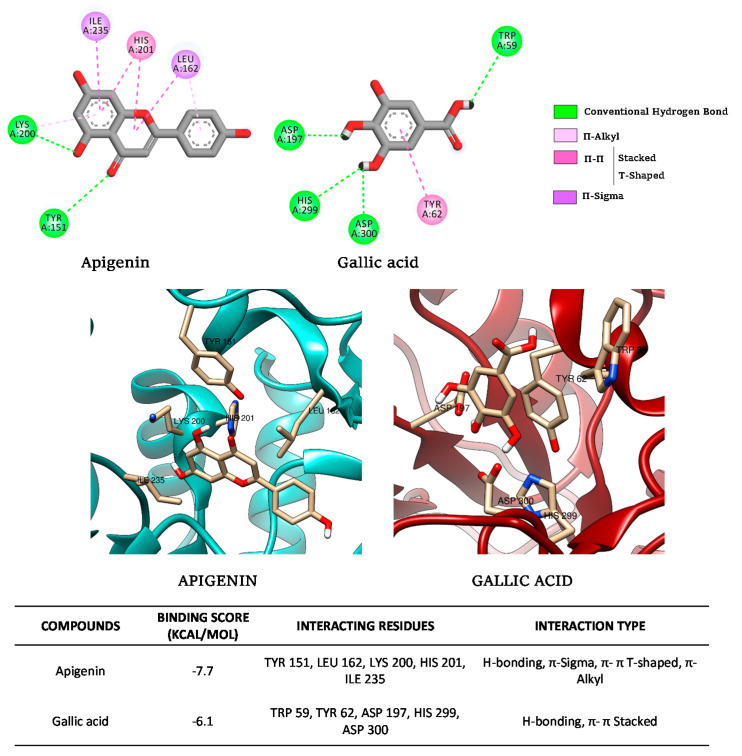
Molecular docking simulations of binding of apigenin and gallic acid to human pancreatic α-amylase.

Figure 9 presents the docking results made for apigenin and gallic acid for their possible binding to pancreatic α-amylase.

These results confirmed the binding of both compounds in the active site of the pancreatic α-amylase thus confirming their competitive action. The calculated affinities were −7.7 kcal/mol and −6.1 kcal/mol for apigenin and gallic acid, respectively, confirming our experimental enzymatic data. This difference in favor of apigenin can be the consequence of additional π–sigma interactions (with I235 and L162 of the enzyme) observed for apigenin in addition to π–π interaction (with H201 for apigenin and Y62 for gallic acid) and conventional hydrogen bonds (with Y200 and Y151 for apigenin, and W59, D197, H299, and E300 for gallic acid). Similar residues, interactions modes, and affinity values were identified for competitive binding of different polyphenols in the active site cavity of α-amylase [11,12].

### 3.3. Evaluation of N. sativa Extracts/Fractions Toxicity in Mice

**Figure 10:**
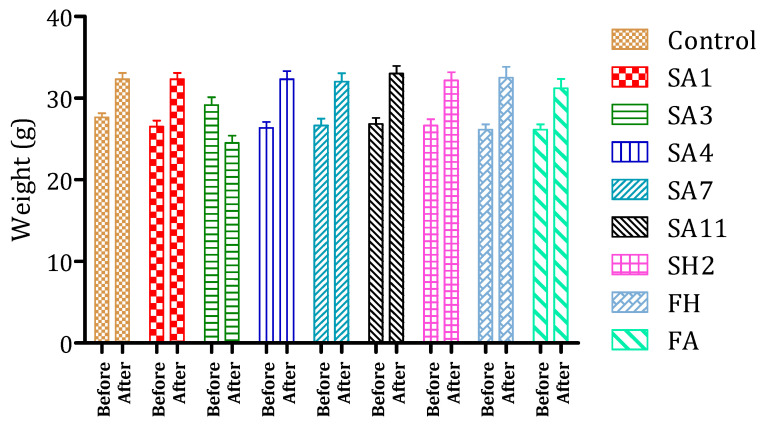
Toxicity evaluation by weight comparison of mice before and after administration of the hexane (EH) and acetone (EA) mother extracts and their fractions.

The toxicity of *N. sativa* seed extracts and fractions was investigated (Figure 10). The fractions did not present any toxicity signs at used doses such as mortality, diarrhea, or abnormal mobility. Only a fraction (SA3) caused some toxicity signs such as hyperactivity, abnormal mobility, and weight loss (10%). In the case of the other fractions, they caused significant mice weight gain of 18% (Figure 9). These results reinforce the interest in *N. sativa* seed extracts for their possible use as nutraceuticals in the prevention of T2DM. Several studies have investigated *N. sativa* toxicity. Monitoring of vital functions for 4 weeks of *N. sativa* powder-fed mice at multidoses presented no toxic effects [48]. Other studies have confirmed this result [49,50]. Furthermore, the protective effect of thymoquinone from induced toxicity of cancer cyclophosphamide drugs [51] and of *N. sativa* hexane extract on ethanol toxicity on groups of rats [52] confirmed that *N. sativa* could present a protective effect against hepatotoxicity and kidney toxicity results. These results show the safety of this plant and encourage researchers to supplement and study the effect of these fractions on enzyme activity (α-glucosidase and α-amylase) in vivo.

## 4. Conclusions

*N. sativa* seeds have traditionally been used for a variety of purposes, including potential antidiabetic properties. Moreover, some research on model animals has shown that it affects blood sugar. Diabetes mellitus management demands precise postprandial glycemic control, which can be achieved by inhibiting carbohydrate enzymes. As a result, it is conceivable that the effect of *N. sativa* seeds is related to an inhibitory effect on the digestive enzymes α-glucosidase and α-amylase.

To test this hypothesis, we made different polarity *N. sativa* seed extracts (hexane and acetone) as well as fractions of these extracts and evaluated their potential inhibitors on these two enzymes. The hexane (EH) and acetone (AE) extracts of *N. sativa* seeds, as well as their fractions (8 fractions (SH1–8) from EH and 11 fractions (SA1–11) from AE) obtained by column chromatography, were characterized by GC–MS and HPLC–DAD, resulting in the identification of 63 compounds (48 in hexane extract and 15 in acetone extract), which is the most comprehensive phytochemical characterization of *N. sativa* seeds to our knowledge.

The putative inhibitory activity of each *N. sativa* hexane and acetone extract and fraction against intestinal α-glucosidase and pancreatic α-amylase was then examined. The acetone fraction SA3 containing (−)-catechin and gallic acid inhibited intestinal α-glucosidase to the greatest extent. *N. sativa* extracts were also effective at inhibiting pancreatic α-amylase activity, with the acetone fraction SA2, which was rich in apigenin and gallic acid, being the most effective.

The possible roles of apigenin, (−)-catechin, and gallic acid in the inhibitory activity of *N. sativa* extracts and fractions were confirmed through additional characterization using several methodologies (IC_50_, inhibition modes by examining enzyme kinetic characteristics, and molecular docking). All of these analyses pointed in the same direction, and while they support previous research, here, their inclusion in the same study allows for a more direct comparison of the data.

Considering the substantial gastrointestinal adverse effects of acarbose, the current promising therapeutic alternative is to explore natural inhibitors that can supplement or possibly replace this drug to reduce these side effects. Here, we demonstrated that (−)-catechin can act in synergy with acarbose to inhibit the α-glucosidase enzyme, whereas apigenin showed an additive effect with acarbose to inhibit the α-amylase enzyme. In that context, the current results are particularly significant.

Finally, toxicity assay revealed that *N. sativa* extracts are nontoxic up to a concentration of 10 g/kg, allowing them to be evaluated further for future potential applications, in vivo.

Altogether our results support the traditional use of *N. sativa* seeds to treat DM, suggest a new inhibiting action on digestive enzymes that may support its hypoglycemic impact, and suggest that *N. sativa* extracts, fractions, and/or derived chemicals may be promising for the prevention and treatment of T2DM.

## Figures and Tables

**Table 1:** Kinetic parameters of intestinal α-glucosidase and pancreatic α-amylase inhibitions.

Ligand	Km (mM)	Vmax (µM/min)	Inhibition Type
**intestinal α-glucosidase**			
Control ^1^	427.6 ± 1.3	14.1 ± 0.7	-
(−)-Catechin	121.9 ± 1.8	13.1 ± 0.9	Noncompetitive
Gallic acid	202.8 ± 3.1	14.7 ± 1.2	Noncompetitive
Acarbose	426.6 ± 2.4	23.9 ± 0.5	Competitive
**pancreatic α-amylase**			
Control ^1^	82.7 ± 1.6	29.6 ± 0.8	-
Apigenin	82.8 ± 1.3	90.1 ± 0.7	Competitive
Gallic acid	87.3 ± 2.4	76.9 ± 1.4	Competitive
Acarbose	82.7 ± 1.4	53.8 ± 1.1	Competitive

^1^ Control represents the enzymatic parameters Km and Vmax determined in absence of an inhibitor.

**Table 2:** IC_50_ values for intestinal α-glucosidase and pancreatic α-amylase inhibitions.

Ligand	IC_50_ (µM)
**intestinal α-glucosidase**	
(−)-Catechin	39.09 ± 1.33
Gallic acid	13.40 ± 1.85
Acarbose	1.96 ± 0.18
**pancreatic α-amylase**	
Apigenin	13.91 ± 2.79
Gallic acid	27.79 ± 2.06
Acarbose	3.07 ± 0.03

## Data Availability

All the data supporting the findings of this study are included in this article.

## Supplementary Materials

The following are available online at https://www.mdpi.com/article/10.3390/foods10081818/s1, Figure S1: GC–MS chromatograms of (a) hexane extract (EH, ×1,000,000) and (b) acetone extract (EA, x 100,000) of *N. sativa* seed. Details of compound identifications are provided in Table S1; Figure S2: GC–MS chromatograms of the 8 hexane fractions (SH1–SH8) resulting from the fractionation on silica gel column of the hexane extract (EH) from *N. sativa* seed. Details of compound identifications are provided in Table S1; Figure S3: HPLC–DAD chromatograms of the acetone extract (EA) from *N. sativa* seed and its fractions (SA1–SA11) resulting from its fractionation on silica gel column. Details of compound identifications are provided in Table S1; Figure S4: UV spectra of the compounds from acetone extract (EA) from *N. sativa* seed identified by HPLC–DAD analysis; Table S1: Characteristics and relative abundance (% of TIC) of compounds from *N. sativa* hexane extract (EH) and its fractions (SH1–SH8) and acetone extract (EA) identified by GC–MS analysis; Table S2: Characteristics and relative abundance (% of peaks relative area) of compounds from *N. sativa* acetone extract (EA) and its fractions (SA1–SA11) identified by HPLC–DAD analysis (by comparison with commercial standards).
