# Supplementary material for: Linking the Phytochemicals and the α-Glucosidase and α-Amylase Enzyme Inhibitory Effects of Nigella sativa Seed Extracts
Source: Foods. 2021 Aug 6;10(8):1818. doi: 10.3390/foods10081818 (PMC8393492; doi:10.3390/foods10081818)
Supplement: Supplementary file 1 [file foods-10-01818-s001.zip › foods-1219660-supplementary.pdf]

Linking the Phytochemicals and the α-Glucosidase and α-Amylase Enzyme Inhibitory Effects of *Nigella Sativa* Seed Extracts

Salima Tiji [1,*], Mohamed Bouhrim [2], Mohamed Addi [3], Samantha Drouet [4], Christophe Hano [4,*], Mohamed Bnouham [2], Mostafa Mimouni [1]

[1] Laboratory of applied Chemistry and Environment (LCAE) Faculty of Sciences Oujda (FSO), University Mohammed First (UMP), Morocco; salimatiji@gmail.com (S.T.); mimouniosrn@gmail.com (M.M.).
[2] Laboratory of Bioresources, Biotechnology, Ethnopharmacology and Health; Faculty of Sciences Oujda (FSO), University Mohammed First (UMP) Morocco; mohamed.bouhrim@gmail.com (M.Bo); mbnouham@yahoo.fr (M.Bn.).
[3] Laboratoire d'Amélioration des Productions Agricoles, Biotechnologie et Environnement, (LAPABE), Faculté des Sciences, Université Mohammed Premier, Oujda, Morocco; m.addi@ump.ac.ma (M.A.).
[4] Laboratoire de Biologie des Ligneux et des Grandes Cultures, INRA USC1328, Orleans University, 45067 Orléans CEDEX 2, France; samantha.drouet@univ-orleans.fr (S.D.) ; hano@univ-orleans.fr (C.H.).
* Correspondence: salimatiji@gmail.com (S.T.); hano@univ-orleans.fr (C.H.)

Supplementary Materials

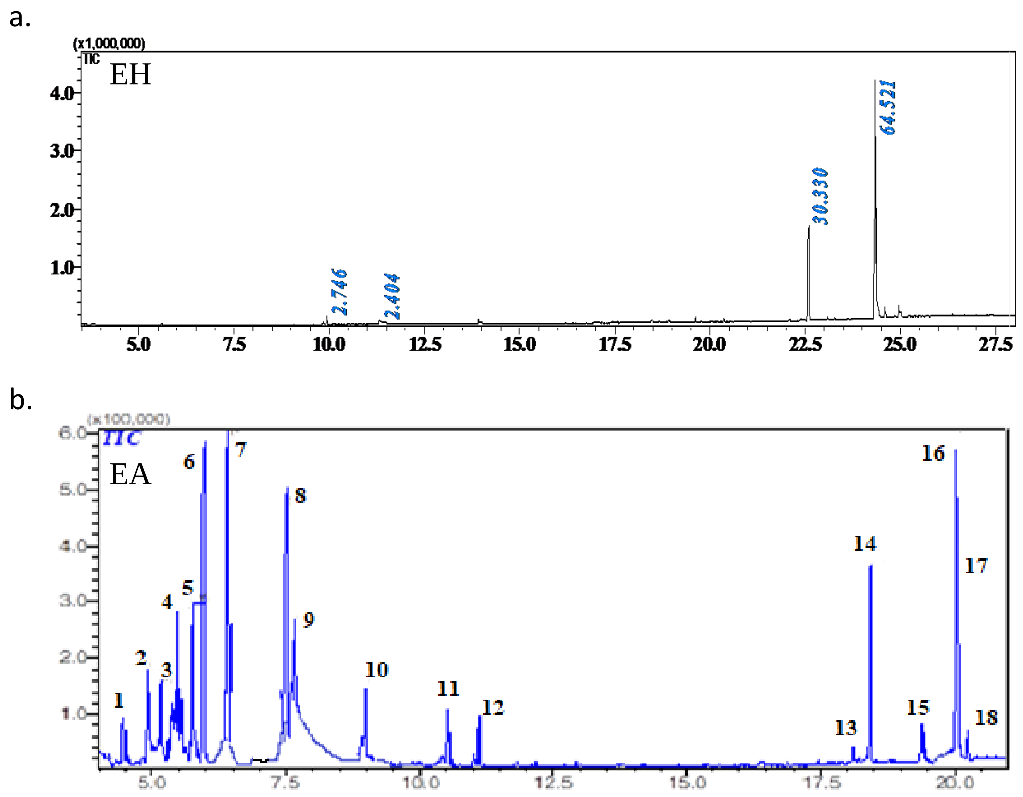

Figure S1: GC-MS chromatograms of **(a)** hexane extract (EH, x 1, 000, 000) and **(b)** acetone extract (EA, x 100, 000) of *N. sativa* seed. Details of compound identifications are provided in Table S1.

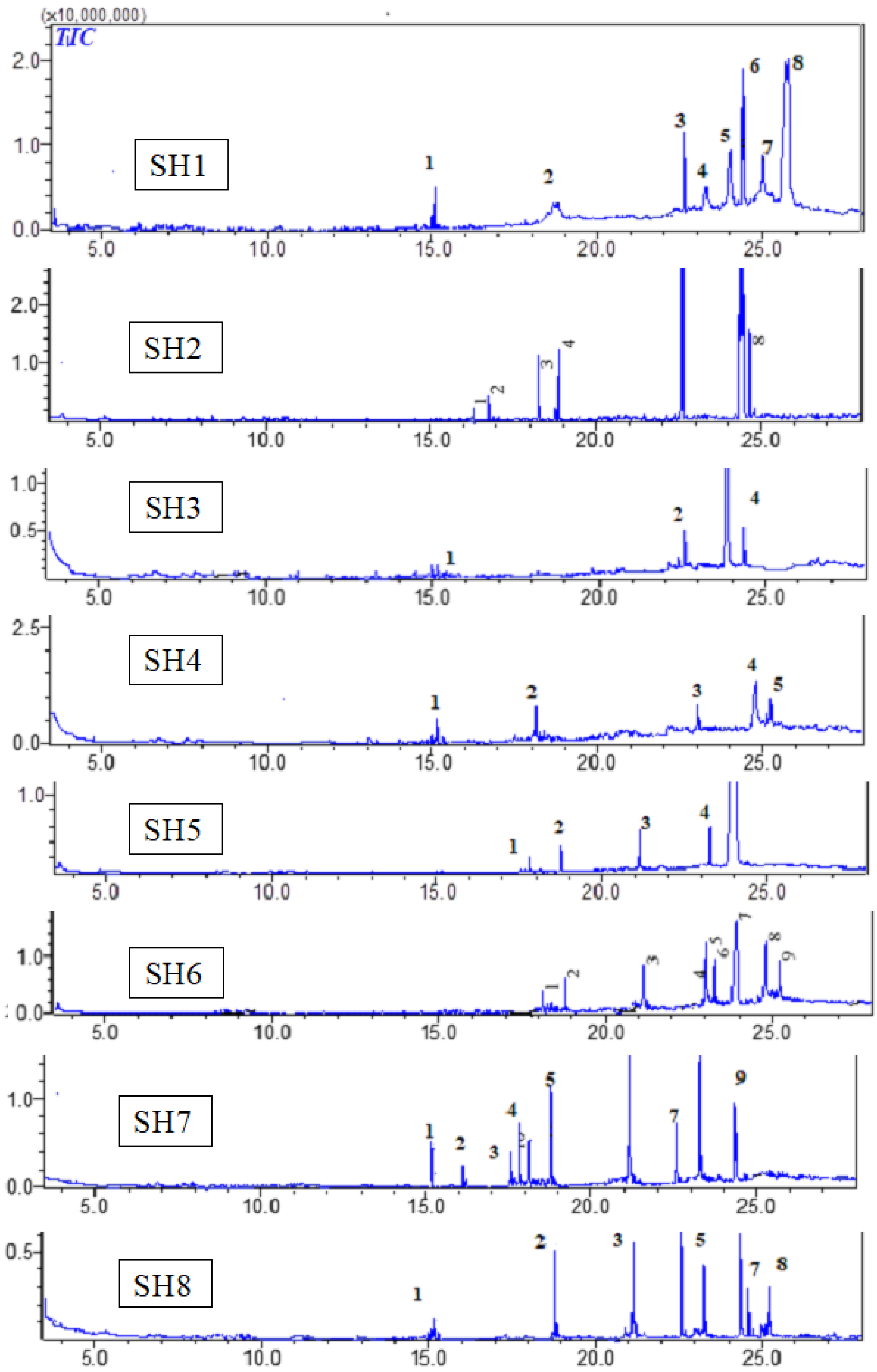

Figure S2: GC-MS chromatograms of the 8 hexane fractions (SH1 to SH8) resulting from the fractionation on silica gel column of the hexane extract (EH) from *N. sativa* seed. Details of compound identifications are provided in Table S1.

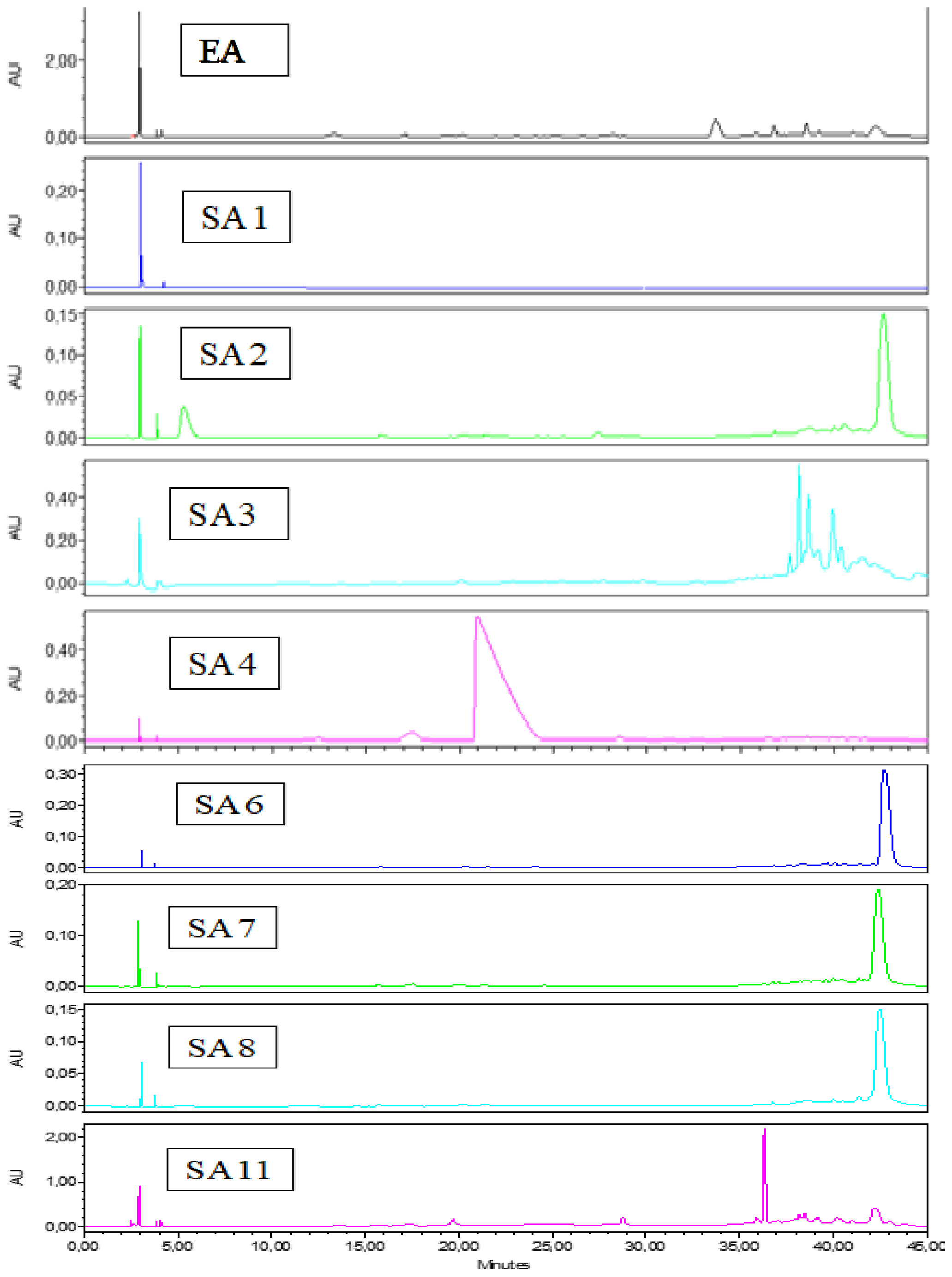

Figure S3: HPLC-DAD chromatograms of the acetone extract (EA) from *N. sativa* seed and its fractions (SA1-SA11) resulting from its fractionation on silica gel column. Details of compound identifications are provided in Table S1.

Compound	Chemical structure	UV spectra
gallic Acid	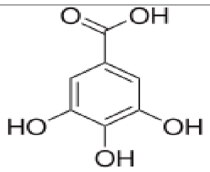	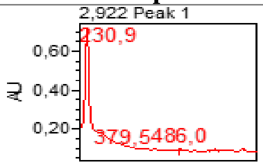
apigenin	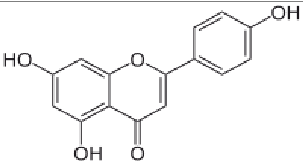	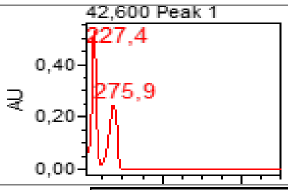
catechin	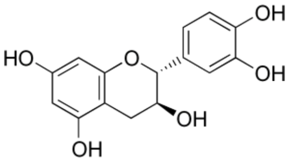	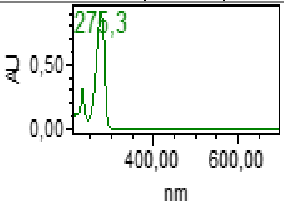
thymoquinone	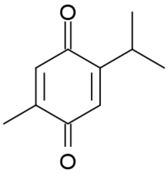	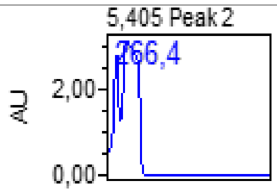
naringenin	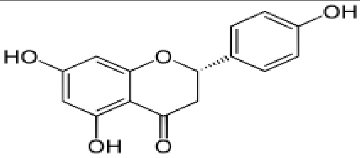	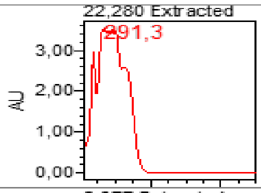
L-ascorbic acid	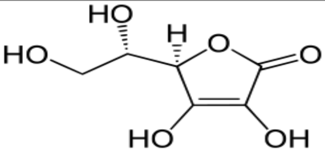	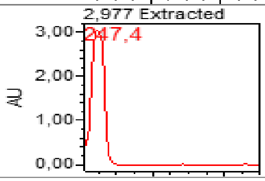
L-cysteine	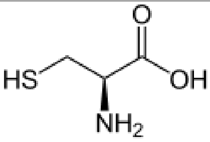	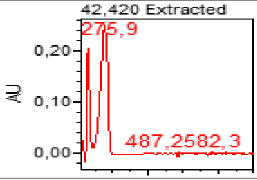
rutin	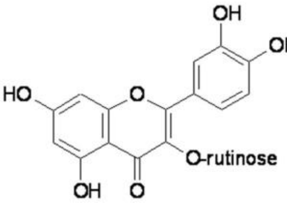	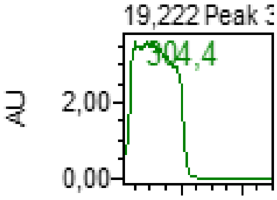

Figure S4: UV spectra of the compounds from acetone extract (EA) from *N. sativa* seed identified by HPLC-DAD analysis.

Table S1: Characteristics and relative abundance (% of TIC) of compounds from *N. sativa* hexane extract (EH) and its fractions (SH1-SH8) and acetone extract (EA) identified by GC-MS analysis.

Name of the compound	PubChem CID	Molecular formula	RT (min)	Relative quantity (%)										
				EH	SH1	SH2	SH3	SH4	SH5	SH6	SH7	SH8	[redacted]	EA
(1,3,+A2:J363-trimethylnonyl)benzene	569261	$C_{18}H_{30}$	5.95											21.62
(2,2-dimethyl-1,3-dioxolan-4-yl)methyl acetate	252333	$C_8H_{14}O_4$	7.66											2.94
(R)-solketal	736056	$C_6H_{12}O_3$	5.14											3.28
(Z)-9-tricosene	5365075	$C_{23}H_{46}$	23.28						2.70	6.60	9.74			
1,2-diacetin	66021	$C_7H_{12}O_5$	11.13											1.88
17-pentatriacontene	5365022	$C_{35}H_{70}$	24.59					60.37						
1-monolinolein	6436630	$C_{21}H_{38}O_4$	20.03											6.85
2,4-ditert-butylphenyl-5-hydroxypentanoate	605777	$C_{19}H_{30}O_3$	17.83								3.87			
2,6,11,15-tetramethylhexadecane	136331	$C_{20}H_{42}$	17.55						0.29		8.44			
2,9-dimethyldecane	517733	$C_{12}H_{26}$	7.50											17.31
2,4-decadienal	5283349	$C_{10}H_{16}O$	15.11		4.95		1.70					3.82		
2-methyl-3-methylene-1-hepten-5-yne	569164	C_9H_{12}	4.92											4.56
2-methyl-heptadecane	15265	$C_{18}H_{38}$	18.13								11.68			
7,9-dimethylhexadecane	545945	$C_{18}H_{38}$	15.18					9.97			6.11			
8-hydroxy-octanoic acid	69820	$C_8H_{16}O_3$	16.23			2.61								
8-methylhexadecane	530042	$C_{17}H_{36}$	15.18								3.71			
8-octadecenoic acid	5282758	$C_{18}H_{34}O_2$	24.43			1.25								
9-eicosene (E)	5365037	$C_{20}H_{40}$	18.78						1.79		16.82	16.53		
9-hexacosene	5363630	$C_{26}H_{52}$	24.24							3.95		8.59		
acetostearin	314293	$C_{23}H_{44}O_5$	11.31	2.40										
azelaic acid	2266	$C_9H_{16}O_4$	18.26			9.44								
beta-cymene	10812	$C_{10}H_{14}$	6.41											15.76
bromopropanoic acid	11729	$C_3H_5BrO_2$	23.26		9.30									
butyl 12-acetoxyoctadecanoate	222097	$C_{24}H_{46}O_3$	25.79		28.12									
cumene	7406	C_9H_{12}	5.46											3.84

dodecanal dimethyl acetal	84559	$C_{14}H_{30}O_2$	18.89			12.14							[redacted]	
dodecane	8182	$C_{12}H_{26}$	8.98										[redacted]	3.56
eicosane	8222	$C_{20}H_{42}$	18.13							2.19			[redacted]	
heptadecanoic acid	10465	$C_{17}H_{34}O_2$	24.64		11.02	16.35							[redacted]	
heptadecyl dichloroacetate	546049	$C_{19}H_{36}Cl_2O_2$	18.77							9.20	6.42	16.84	[redacted]	
heptyl valerate	79544	$C_{12}H_{24}O2$	4.47										[redacted]	2.72
hexadecane	11006	$C_{16}H_{34}$	18.13					10.84					[redacted]	
l-(+)-ascorbic acid 2,6-dihexadecanoate	54722209	$C_{38}H_{68}O_8$	23.03							14.37			[redacted]	
lauric acid	3893	$C_{12}H_{24}O_2$	18.68		1.87								[redacted]	
lignocerol	10472	$C_{24}H_{50}O$	24.79					7.62					[redacted]	
linoleic acid	5280450	$C_{18}H_{32}O_2$	24.36	64.52	18.29	17.10			92.54		14.08		[redacted]	1.12
methyl 9-oxononanoate	74732	$C_{10}H_{18}O_3$	16.77			2.60							[redacted]	
methyl oleate	5364509	$C_{19}H_{36}O_2$	24.39									5.57	[redacted]	
methyl stearate	8201	$C_{19}H_{38}O_2$	24.62									10.49	[redacted]	
methyl-10,14,18,22-tetramethyltricosanoate	545641	$C_{28}H_{56}O_2$	22.58					11.17					[redacted]	
methyl-palmitate	8181	$C_{17}H_{34}O_2$	22.60				5.65		2.65		18.89	24.91	[redacted]	
nonadecanoic acid	12591	$C_{21}H_{42}O_2$	20.26										[redacted]	0.98
oleic acid	445639	$C_{18}H_{34}O_2$	24.34	2.74			5.31			12.34			[redacted]	0.56
palmitic acid	985	$C_{16}H_{32}O_2$	22.61	30.33	8.64	40.98				4.09			[redacted]	7.29
Psi-cumene	7247	C_9H_{12}	5.73										[redacted]	3.23
stearic acid	5281	$C_{18}H_{36}O_2$	18.12										[redacted]	0.73
stearyl alcohol	8221	$C_{18}H_{38}O$	23.58		17.79		87.32			47.22		13.23	[redacted]	
thymol	6989	$C_4H_{14}O$	10.53										[redacted]	1.84

Table S2: Characteristics and relative abundance (% of peaks relative area) of compounds from *N. sativa* acetone extract (EA) and its fractions (SA1-SA11) identified by HPLC-DAD analysis (by comparison with commercial standards).

Name of the compound	PubChem CID	Molecular formula	RT (min)	λmax (nm)	Relative quantity (%)								
					EA	SA1	SA2	SA3	SA4	SA6	SA7	SA8	SA11
apigenin	5280443	$C_{15}H_{10}O_5$	13.27	337	13.27		73.50						
gallic acid	370	$C_7H_6O_5$	2.92	369	23.5	98.90	25.80	5.36					
kaempferol	5280863	$C_{15}H_{10}O_6$	32.54	365	2.03								
l-ascorbic acid	54670067	$C_6H_8O_6$	2.97	295	2.03						13.90		
l-cysteine	5862	$C_3H_7NO_2S$	42.42	320	13.2						82.17		
l-histidine	6274	$C_6H_9N_3O_2$	42.24	300									12.30
naringenin	932	$C_{15}H_{12}O_5$	22.28	292	8.45				88.70				
catechin	73160	$C_{15}H_{14}O_6$	39.92	250				79.83					
quercetin	5280343	$C_{15}H_{10}O_7$	27.74	357	8.42								7.65
rutin	5280805	$C_{27}H_{30}O_{16}$	19.22	360	4.95								4.80
thymoquinone	10281	$C_{10}H_{14}O_2$	5.40	289	3.82		12.30						
ND1	-	-	3.74	254	4.85					10.74			
ND2	-	-	3.80	300	3.65							25.21	
ND3	-	-	42.50	383	3.73							72.01	
ND4	-	-	42.73	280	8.05					86.72			
